# A novel method to determine linac mechanical isocenter position and size and examples of specific QA applications

**DOI:** 10.1002/acm2.13257

**Published:** 2021-05-30

**Authors:** Jacek M. Chojnowski, Jonathan R. Sykes, David I. Thwaites

**Affiliations:** ^1^ Mid North Coast Cancer Institute Coffs Harbour Health Campus Coffs Harbour NSW Australia; ^2^ Institute of Medical Physics School of Physics University of Sydney Sydney NSW Australia; ^3^ Department of Radiation Oncology Blacktown Cancer & Haematology Centre Blacktown NSW Australia

**Keywords:** mechanical isocenter, quality assurance, radiation isocenter, WL test

## Abstract

The most important geometric characteristic of stereotactic treatment is the accuracy of positioning the target at the treatment isocenter and the accuracy of directing the radiation beam at the treatment isocenter. Commonly, the radiation isocenter is used as the reference for the treatment isocenter, but its method of localization is not strictly defined, and it depends on the linac‐specific beam steering parameters. A novel method is presented for determining the linac mechanical isocenter position and size based on the localization of the collimator axis of rotation at arbitrary gantry angle. The collimator axis of rotation position is determined from the radiation beam center position corrected for the focal spot offset. The focal spot offset is determined using the image center shift method with a custom‐design rigid phantom with two sets of ball‐bearings. Three specific quality assurance (QA) applications and assessment methods are also presented to demonstrate the functionality of linac mechanical isocenter position and size determination in clinical practice. The first is a mechanical and radiation isocenters coincidence test suitable for quick congruence assessment of these two isocenters for a selected energy, usually required after a nonroutine linac repair and/or energy adjustment. The second is a stereotactic beam isocentricity assessment suitable for pretreatment stereotactic QA. The third is a comprehensive linac geometrical performance test suitable for routine linac QA. The uncertainties of the method for determining mechanical isocenter position and size were measured to be 0.05 mm and 0.04 mm, respectively, using four available photon energies, and were significantly smaller than those of determining the radiation isocenter position and size, which were 0.36 mm and 0.12 mm respectively. It is therefore recommended that the mechanical isocenter position and size be used as the reference linac treatment isocenter and a linac mechanical characteristic parameter respectively.

## 1 INTRODUCTION

Quality control guideline TG142^1^ recommends that the coincidence of radiation and mechanical isocenters, as well as imaging and treatment isocenters, should be verified annually. The assessment of coincidence of radiation and mechanical isocenters is difficult to perform directly since the mechanical isocenter is traditionally determined using gantry and couch‐mounted mechanical pointers^2^ and graph paper, and the radiation isocenter is determined by means of the Winston‐Lutz (WL) test^3^ with a ball‐bearing (BB) phantom, historically with radiosensitive film and nowadays with the electronic portal imaging detector (EPID).^4^ In the WL test, the BB phantom, representing a target, is attached to the patient support table and aligned to the treatment isocenter. EPID images of the BB phantom are then acquired with the collimated beam at various gantry and collimator angles and analysed in terms of deviations of the radiation field centers from the BB phantom (i.e. beam isocentricity).

Liu at al.^5^ presented an improved version of the mechanical and radiation coincidence test, using a special EPID (BIS 710) sensitive to light and radiation that significantly reduces the measurement uncertainty. However, the mechanical isocenter was assumed to be represented by the optical isocenter (light and crosshair system), which might not be the case. An alternative and less complex method is to utilize the laser system (used for initial patient positioning) as the reference coordinate frame. In that case lasers are first aligned to the mechanical pointer adjusted to represent the mechanical isocenter. Rosca et al.^6^ presented a high accuracy method of verifying the laser system alignment with the radiation isocenter using phosphor plates. The method also provides characteristics of the radiation isocenter geometry, similar to that achieved with the commonly used WL test and ball‐bearing phantom. However, the method is still relatively time and resource intensive.

Letourneau et al.^7^ discussed how to assess accurately the alignment of the radiation beam axis with the three independent mechanical axes of rotations of the collimator, the gantry and the couch based on an analytical model, custom‐made phantoms and analysis software. However, the mechanical isocenter location was not defined, and therefore the coincidence of radiation and mechanical isocenters could not be unambiguously determined.

Zhang et al.^8^ considered carefully the issues related to having various linac isocenters, commonly referred to as those for mechanical, radiation and imaging systems, depending on how the isocenter position is determined. They presented a theoretical framework of the linac isocenter starting from fundamental concepts of a definition expressed mathematically. They also recommended that the linac mechanical isocenter, called in brief ‘linac isocenter’, should be used as the reference isocenter and that the other isocenters should be aligned with it, including the treatment isocenter. Zhang et al.^8^ asserted that there should be one ‘center of collimation’, that is common for all Beam Limiting Devices (BLD) (e.g. MLC, diaphragms, cones) since all BLDs can be calibrated against this reference ‘center of collimation’. This approach is only effective when the focal spot position (point of the electrons hitting the target) is aligned with the collimator axis of rotation i.e. the focal spot offset (FSO) is zero.

The effect of FSO was first reported by Lutz et al.^9^ They noticed that a transverse beam spot offset caused the radiation field positions to shift laterally, which resulted in two radiation fields at two opposed gantry angles to be misaligned (‘FSO effect’).

Sonke et al.^10^ and Slama et al.^11^ demonstrated that the focal spot position for both Elekta and Varian linacs is changing at beam start‐up and hence depends on the MU delivered (i.e., beam on time). On average the beam position stabilizes after 10 MU for Truebeam linacs and about 20 MU for Elekta and older design (Trilogy, iX) Varian linacs.

Nyiri et al.^12^ considered the dependency of the x‐ray shadows of radio opaque rods on the distance of the rod from the radiation source. They observed that when the focal spot is not aligned with the collimator axis of rotation the distance between the two centers of the images formed by projected x‐ray shadows of two rods positioned at different distances from the radiation source is dependent on the collimator angle. They proposed the image center shift method to correlate geometrically the FSO (D_FSO_) and the distance between the two image centers (Δ_FSO_). The centers of the images were calculated as the average position of projected x‐ray shadows of the two rods, respectively (placed at distances d_1_ and d_2_ from the radiation source), while rotating the collimator, and were measured using the EPID (placed at a distance d_EPID_ from the radiation source):

(1) $$\Delta_{\text{FSO}}/D_{\text{FSO}}=\left(d_{\text{EPID}}/d_{2}‐d_{\text{EPID}}/d_{1}\right)$$

In practice, this means that the FSO effect causes the difference between radiation field centers to vary depending on the FSO value, the position of the BLD forming the field aperture and the position of the measurement plane (i.e., EPID). Chojnowski et al.^13^ explored the FSO effect for a clinically relevant example of the Elekta Agility MLC calibration procedure, where the reference calibration position is determined using two BLDs (i.e., the MLC and diaphragms) that are physically at different distances from the radiation source. They reported a correlation factor of 0.7 between the MLC calibration error and the FSO scaled back from the EPID level to the isocenter level, i.e., if the FSO is 0.2 mm, it results in an MLC miscalibration of 0.14 mm.

The FSO effect is larger when the difference between distances of the BLDs to the radiation source is larger. The correlation factor between the WL test and the FSO for the Elekta linac with the Agility head is approximately 1.8 (see Eq. (1), [d_EPID_/d_2(MLC)_ – d_EPID_/d_1(BB)_] * [d_ISO_/d_EPID_]; d_EPID_ = 160 cm (EPID positioned at 160 cm from the radiation source), d_1(BB)_ = 100 cm (ball‐bearing positioned at the isocenter), d_2(MLC)_ = 35.5 cm (MLC positioned at 35.5 cm from the radiation source), d_ISO_ = 100 cm (scaling back to the isocenter)). If the radial^1^ FSO for an optimized beam on Elekta linacs is about 0.2–0.3 mm,^13^, ^14^ this would result in about 0.4–0.5 mm shift of the radiation isocenter longitudinally^1^ from the mechanical isocenter. Therefore, nowadays, the radiation isocenter is often used as the treatment isocenter, not the mechanical isocenter as in the past, and the two major linac manufacturers (Elekta and Varian) use it in their proprietary procedures to calibrate (align) the imaging systems’ isocenters. This approach does not, however, eliminate the FSO effect as such, but just minimizes it. If stereotactic treatments are performed with cones, the treatment isocenter defined by the MLC and diaphragms is different compared to the radiation isocenter collimated by the cone due to the FSO effect, as will be illustrated in the Methods and Results below.

To eliminate the FSO effect, the authors expanded the theoretical Zhang et al.^8^ concept of the ‘center of collimation’ to be more practical and include the requirement for the FSO to be zero (i.e., include the FSO correction). Therefore, the concept of the ‘central axis of collimation’ is proposed here (referred to as the collimator CAX), commonly known as the collimator axis of rotation, and also that all radiation beam collimation axes (referred to here as the beam CAX) as well as all BLDs should be aligned with it.

The collimator CAX position is very reproducible^15^ and the authors agree with the recommendation of Zhang et al.^8^ that all radiation and imaging isocenters should be aligned to the stable mechanical isocenter.^16^ However, there are no published methods describing how to position the BB phantom precisely at the mechanical isocenter so that it can be used to assess the alignment of linac isocenters utilizing a common WL test.

Nyiri et al.^12^ presented the image center shift method, as mentioned above, to measure the FSO and to localize the collimator CAX at the EPID level with a jig comprising two rods, however, the procedure can only be used at gantry angle 0° due to the nonrigid design of the jig. Riis et al.^14^ designed a special rigid phantom with two BBs that is conceptually similar to Nyiri’s jig but can be used at any gantry angle. However, neither the BB phantom nor the EPID can be placed at the linac isocenter due to the collision risk with the bulky phantom.

This study expands the work of Nyiri et al.^12^ and Riis et al.^14^ to localize the collimator CAX using radiation, utilizing a phantom of a different design and a modified method to guide the BB phantom to the mechanical isocenter. The mechanical isocenter position is defined in this study using the ‘collimator axis trajectory’ approach proposed by Skworcow et al.,^16^ which means that the mechanical pointer axis is utilized, instead of the mechanical pointer end, where the latter is common in standard approaches. It is important to emphasize that differences between mechanical isocenters determined using ‘collimator axis trajectory’ and standard approaches may be over 1 mm. The ‘collimator axis trajectory’ approach is more clinically relevant and conceptually similar to the radiation isocenter determination approach.

In this work, a method is proposed to localize the collimator CAX and the mechanical isocenter using radiation and was validated against varying radiation beam parameter settings of energy and MU. Based on the findings, three examples of procedures for specific linac quality assurance (QA) assessments were developed and presented, namely:

- *Coincidence* assessment of mechanical and radiation isocenters
- *Stereotactic* beam isocentricity assessment
- *Comprehensive* assessment of linac geometrical performance

## 2 METHODS AND MATERIALS

### 2.1 Linear accelerator

Measurements were carried out on two Elekta Versa HD linear accelerators (Elekta AB, Stockholm, Sweden) using 6MV and 15MV high energy photon beams with flattening filter (WFF) and 6MV and 10MV high energy flattening filter free (FFF) photon beams. FFF beams were not in clinical use at the time of testing and were not optimized in terms of the focal spot position. The linacs were equipped with the iViewGT megavoltage (MV) portal imaging system, with the MV EPID panel consisting of 1024 × 1024 pixels (pixel size is 0.4 mm), and the Elekta X‐ray Volume Imaging (XVI) system for kilovoltage (kV) diagnostic imaging, with the kV EPID panel consisting of 512 × 512 pixels (pixel size is 0.8 mm). The MV EPID panel was positioned at the nominal distance of 160 cm from the radiation source. The linacs were equipped with the Elekta Precise treatment table used for patient treatment support and positioning.

The linacs could also be equipped with the add‐on Aktina (Aktina Medical Corporation, Congers, NY, USA) cones (circular small field collimators) for precise photon beam collimation required for specialized treatment techniques such as stereotactic radiosurgery (SRS) or stereotactic body radiotherapy (SBRT). In this study, one cone size of 27 mm diameter was used.

### 2.2 Phantoms

#### 2.2.1 BB phantom

The BB phantom was a simple phantom originally designed for the WL test with a stainless‐steel ball bearing (denoted here as the BB_WL_), 8 mm in diameter, attached to the end of a polymethyl methacrylate (PMMA) rod.

#### 2.2.2 FSO phantom

**Fig. 1:**
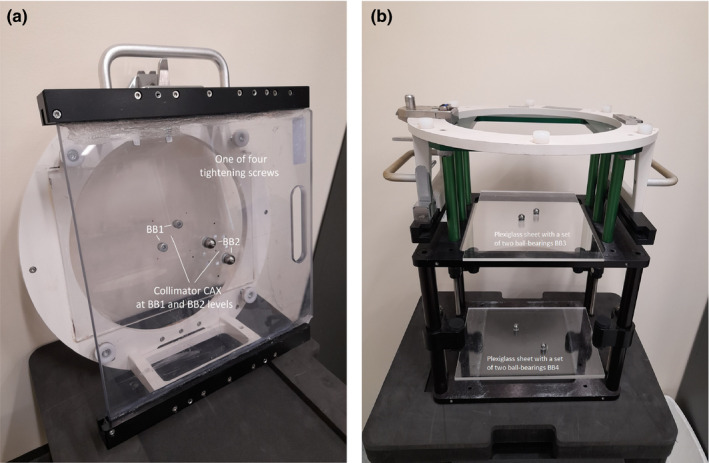
Pictures of (a) the FSO phantom and (b) the Validation phantom with Plexiglas sheets, each contains a set of two ball‐bearings.

The FSO phantom was a specially designed and constructed phantom used for determination of the FSO [see Fig. 1(a)]. It consisted of two Plexiglas sheets attached to the top and bottom mount of an Elekta accessory attachment. Each sheet had two 12 mm diameter ball bearings positioned 2 cm from the collimator CAX and diagonally opposing each other.

Four tightening screws were added to the FSO phantom and were used to remove any small gaps between the linac collimator face plate and the FSO phantom. This and the very rigid construction of the phantom eliminate any possible sag or flex of the phantom as the gantry rotates. The positions of the upper and lower BB sets were at nominal distances to the radiation source of 55 cm and 65 cm (named BB_1_ and BB_2_ sets respectively).

**Fig. 2:**
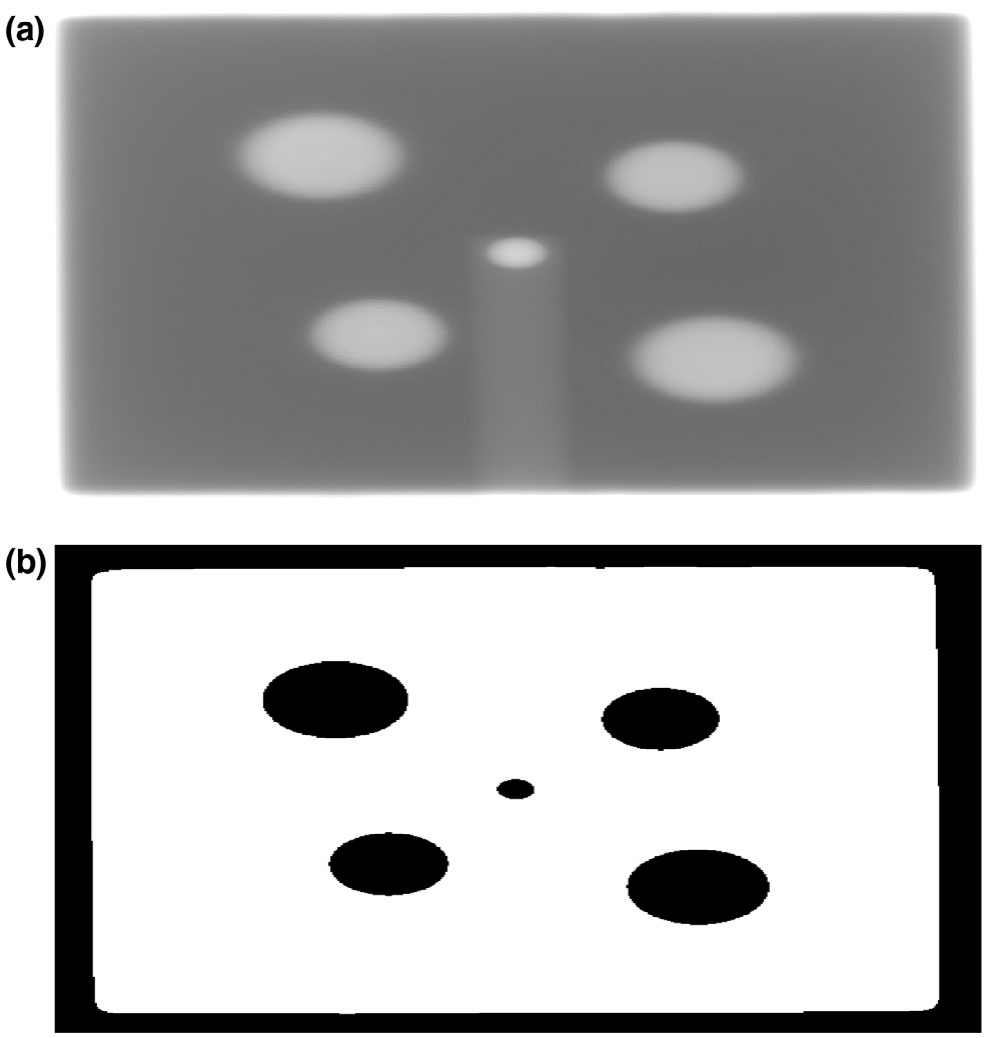
An example of one EPID image (a) acquired for the comprehensive assessment and (b) processed by the IHD software. One central circle represents the BB phantom and four peripheral circles represent the FSO phantom. Two larger peripheral circles are related to the BBs located closer to the radiation source i.e. BB_1_ set, and correspondingly two smaller peripheral circles are related to the BBs located further from the radiation source i.e. BB_2_ set.

The reason for having two BBs on each sheet was to minimize the effect of collimator angle calibration error i.e. any collimator angular error affected both BBs located opposite to each other in regard to the collimator CAX, therefore their combined average position should be unaffected, which would not be the case for only one BB. Four BBs on two sheets were positioned diagonally away from the collimator CAX and the linac isocenter, so that the BB phantom and the FSO phantom can be used at the same time and the acquired EPID images can be automatically processed [see Figs. 2(a) and 2(b)].

#### 2.2.3 Validation phantom

The validation phantom [see Fig. 1(b)] was designed to validate the FSO and the collimator CAX determination methods at gantry 0°. The phantom was similar in design to the FSO phantom, however, did not have four tightening screws and two BB sets were positioned at nominal distances to the radiation source of 70.5 cm and 100 cm (named BB_3_ and BB_4_ sets respectively).

### 2.3 Image processing and analysis software

An in‐house developed (IHD) software was used for processing the acquired EPID images and determining linac isocenters characteristics as well as analysis and graphical presentation of results. The software was written in the MATLAB programming language and software environment (MathWorks, Natick, MA, US). The image processing algorithm initially preprocessed all EPID acquired images to minimize noise and eliminate dead pixels. Then radiation fields and all BBs were detected based on the global thresholding method.

### 2.4 Localizing collimator CAX

#### 2.4.1 Localizing collimator CAX at the EPID level

**Fig. 3:**
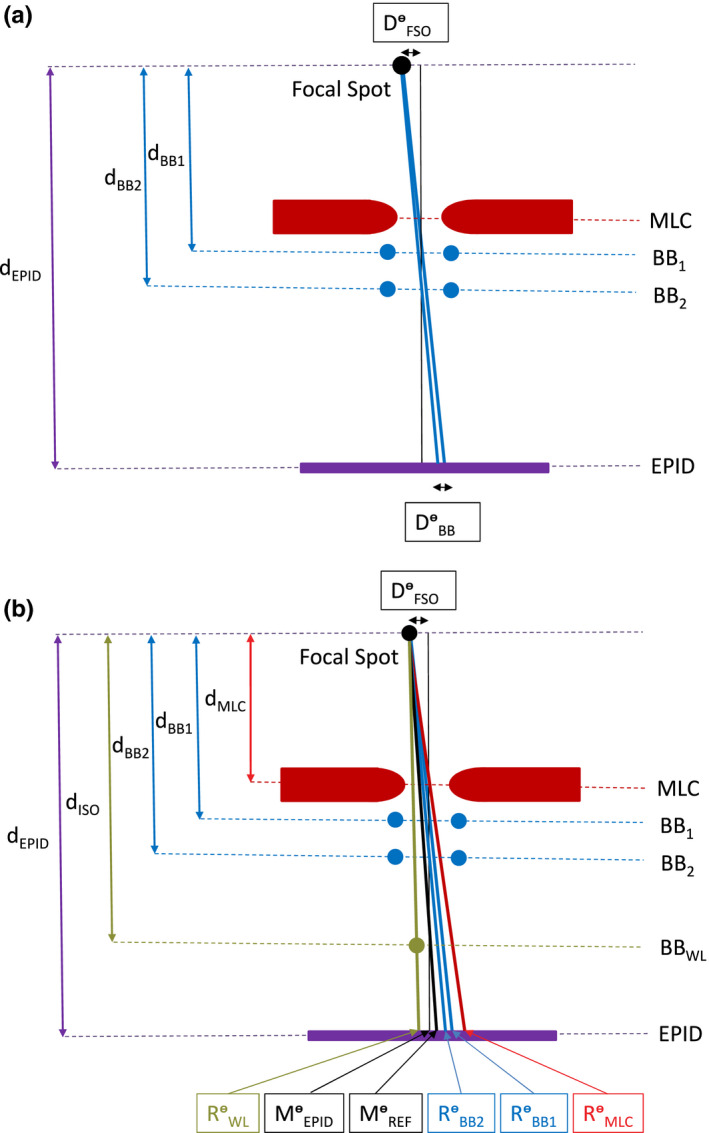
Schematic (not to scale) of the BLDs and the BBs locations and illustration of the concept of (a) determining the focal spot offset (Dᶱ_FSO_) and (b) localizing mechanical collimator CAX (Mᶱ_EPID_) at the EPID level and the reference mechanical collimator CAX (Mᶱ_REF_) at the isocenter level for a given gantry angleᶱ measured by the EPID (for simplicity the diaphragms, the Aktina cone, the BB_3_ and the BB_4_ sets are not included, Rᶱ_MLC_ ‐ position of the radiation beam CAX collimated by the MLC at the EPID level and for gantry angleᶱ; Rᶱ_WL_, Rᶱ_BB2_, Rᶱ_BB2_ ‐ positions of the radiation beam CAX defined by the BB phantom and FSO phantom i.e. BB_1_ and BB_2_ sets, respectively, at the EPID level and for gantry angleᶱ).

The FSO (Dᶱ_FSO_) was determined from the measured distance (Dᶱ_BB_) between beam central axis positions defined by ball‐bearing sets BB_1_ and BB_2_ at any given gantry angleᶱ and distances of both the BB_1_ and the BB_2_ sets and the EPID from the radiation source [see Fig. 3(a)]:

(2) $$D{ᶱ}_{\text{FSO}}=\frac{D{ᶱ}_{\text{BB}}}{\left(d_{\text{EPID}}/d_{\text{BB2}}‐d_{\text{EPID}}/d_{\text{BB1}}\right)}$$

Where: ᶱ‐ gantry angle. Dᶱ_FSO_ ‐ vector of the focal spot offset determined at the radiation source level and for gantry angleᶱ. Dᶱ_BB_ ‐ vector between positions of the beam CAX defined by ball‐bearing sets BB_1_ and BB_2_ measured at the EPID level and for gantry angleᶱ. d_EPID_ = distance of the EPID from the radiation source: 160 cm. d_BB1_ = distance of the ball‐bearing set BB_1_ from the radiation source: 55 cm. d_BB2_ = distance of the ball‐bearing set BB_2_ from the radiation source: 65 cm.

The position of the collimator CAX (Mᶱ_EPID_) at any given gantry angle at the level of the EPID [see Fig. 3(b)] was defined as the radiation beam CAX positions determined by either the ball‐bearing sets BB_1_ (Rᶱ_BB1_) or BB_2_ (Rᶱ_BB2_), at that gantry angle and at the level of the EPID, and the FSO (Dᶱ_FSO_) using the following relationship:

(3) $$M{ᶱ}_{\mathrm{EPID}}=R{ᶱ}_{\text{BB1}}‐D{ᶱ}_{\text{FSO}}\left(1‐\frac{d_{\text{EPID}}}{d_{\text{BB1}}}\right)=R{ᶱ}_{\text{BB2}}‐D{ᶱ}_{\text{FSO}}\left(1‐\frac{d_{\text{EPID}}}{d_{\text{BB2}}}\right)$$

Where: Mᶱ_EPID_ ‐ position of the mechanical collimator CAX at the EPID level and for gantry angleᶱ. Rᶱ_BB1_ ‐ position of the radiation beam CAX defined by the ball‐bearing set BB_1_ at the EPID level and for gantry angleᶱ. Rᶱ_BB2_ ‐ position of the radiation beam CAX defined by the ball‐bearing set BB_2_ at the EPID level and for gantry angleᶱ.

#### 2.4.2 Localizing collimator CAX for the BB phantom

If the BB phantom is positioned at the collimator CAX and at the isocenter level, the radiation beam CAX defined by this BB phantom (Rᶱ_WL_) does not coincide with the collimator CAX as measured by the EPID (Mᶱ_EPID_) at the EPID level [see Fig. 3(b)], due to the FSO effect. Therefore, to assess alignment of the BB phantom to the collimator CAX at the linac isocenter level, the reference position of the collimator CAX (Mᶱ_REF_) should be used [see Fig. 3(b)] that is corrected for the FSO effect and can be calculated as:

(4) $$M{ᶱ}_{\text{REF}}=M{ᶱ}_{\mathrm{EPID}}+D{ᶱ}_{\text{FSO}}\left(1‐\frac{d_{\text{EPID}}}{d_{\text{ISO}}}\right)$$

Where: Mᶱ_REF_ ‐ position of the reference mechanical collimator CAX at the EPID level and for gantry angleᶱ. d_ISO_ = distance of the ball‐bearing BB_WL_ from the radiation source: 100 cm.

### 2.5 Validation

The FSO measurement method [see Eq. (2)] was independently validated using the Validation phantom with the two ball‐bearing sets BB_3_ and BB_4_.

The collimator CAX position determination method at the EPID level [see Eq. (3)] was validated using the four different energies (6MV, 15MV, 6MV FFF, and 10MV FFF) and five different CAX‐defining systems using BLDs and phantoms (MLC only, diaphragms and MLC, the FSO phantom, the Validation phantom and the Aktina cone) in separate executions to observe the deviation of the beam CAX from the collimator CAX, named here as ‘the beam CAX offset’ (BO). A square field size of 12 cm × 12 cm was used (4 cm × 4 cm for the Aktina cone) with 50 MU per field. 50 MU settings per field was considered a reasonable compromise between the minimum MU required for the FSO to be stable^10^ and minimizing overall time of beam delivery for QA purposes. The test was performed at gantry 0°, to avoid MLC and diaphragms sag, and at 13 collimator angles in increments of 30° starting from −180° and ending at +180°. Additionally, the validation test was repeated for the 6MV energy with the FSO phantom with MU settings (5, 10, 20, and 50 per field).

The reference collimator CAX position determination method, at the isocenter level [see Eq. (4)], was validated using the Validation phantom with the ball‐bearing set BB_4_ specifically positioned 100 cm from the radiation source. The reference mechanical collimator CAX position (Mᶱ_REF_), determined using the FSO phantom, should be at the radiation beam CAX position defined by the ball‐bearing set BB_4_ (Rᶱ_BB4_) for any beam energy, since the FSO does not affect the radiation beam CAX position at the isocenter level (i.e., 100 cm from the radiation source), but does at other levels, including the EPID level.

### 2.6 Methods of assessment of linac mechanical, radiation, and imaging isocenters

Based on the presented methods of localizing the collimator CAX and the reference collimator CAX, three assessment procedures were developed, as examples, to address specific linac QA requirements:

- *Coincidence assessment of mechanical and radiation isocenters* for non‐routine linac QA after adjusting a selected energy
- *Stereotactic beam isocentricity* *assessment* for pretreatment stereotactic checks
- *Comprehensive assessment of linac* *geometrical performance* for routine (e.g., monthly) linac QA

All relative positions determined in these assessments were referenced to the position of the mechanical isocenter as the recommended reference linac isocenter in the three‐dimensional linac coordinate system (IEC 1217). All distances measured at the level of the EPID were then scaled back to the linac isocenter level.

#### 2.6.1 Coincidence assessment of mechanical and radiation isocenters

The most efficient and effective way of determining radiation isocenter is by determining beam centers using two opposite collimator angles at each of four cardinal gantry angles.^17^ This approach was used in this study as well, however, there are two gantry angles i.e., −180° and +180° that are opposite the gantry angle 0° and they might have different FSO and BO values, so EPID images were acquired and analysed at both available gantry angles.

Ten megavoltage (MV) images were acquired in total for each tested energy at five gantry angles (±180°, ±90°, and 0°) and two collimator angles (±90°) with 50 MUs (named in this study as the FSO sequence, since the FSO phantom was used). For each acquired EPID image, the IHD software determined both the beam CAX and the collimator CAX.

The radiation isocenter is determined as the centroid of a sphere comprising all beam central axes. The mechanical isocenter in this study is defined as the centroid of a sphere comprising collimator axis of rotation at all gantry angles. Therefore, in theory, the mechanical isocenter definition is similar to the radiation isocenter definition and both isocenters coincide with each other when the beam CAX is aligned with the collimator CAX [i.e., the BO is zero when the FSO is zero; see Eq. (2)] at all gantry angles. The mechanical isocenter position can be determined using the procedure of determining the radiation isocenter position with the beam CAX being corrected for the non‐zero FSO, as described in the localizing collimator CAX section 2.D [see Eq. (3)]. The FSO for each cardinal gantry angle was determined from the EPID images acquired in the same predefined field sequence as the radiation isocenter and analysed by the IHD software.

The coincidence assessment was performed for a 6MV beam in terms of the FSO and the BO at five cardinal gantry angles (−180°, …, +180°).

#### 2.6.2 Stereotactic beam isocentricity assessment

The stereotactic assessment was performed by comparing the relative position of the stationary reference BB phantom to the beam central axes. The BB phantom was placed at the treatment isocenter using the image guided radiotherapy (IGRT) system commissioned for stereotactic treatments. Stereotactic field sequences might have multiple gantry and couch angle combinations, but here the FSO sequence was used for simplicity with the stereotactic 6MV FFF beam. The radiation beam can be collimated by the stereotactic BLD, either by the Aktina cone, as used in this study, or an add‐on MLC attached to the linac head or just the in‐built MLC. Results were reported as the beam offset (BO), collimated by the Aktina cone, the ball‐bearing offset (BBO) and complemented with the traditional beam isocentricity (BI). This method fundamentally performed beam isocentricity assessment and separated it into two independent parameters of the BO and the BBO.

#### 2.6.3 Comprehensive assessment

Comprehensive assessment of linac mechanical, radiation, and (additionally) imaging isocenters is possible by comparing relative positions of the stationary reference BB phantom to individually determined linac mechanical, radiation, and imaging isocenters. Those relative positions are then normalized to a reference point, which is the average of mechanical isocenter position determined for all four energies.

The BB phantom was placed at the treatment isocenter using the IGRT system, which, in this study was the Cone Beam Computed Tomography (CBCT). The alignment of the BB phantom with the mechanical isocenter was reported, which characterizes the overall performance of the IGRT system. The radiation and mechanical isocenters determinations were described in the coincidence assessment, however, in this test all available energies were used.

The imaging isocenter in this study was defined for both MV imaging system (beam and panel) and CBCT (kV) system (beam and panel) as the centroid of the sphere comprising MV or kV image centers at all gantry angles.

The kV imaging system uses a kV radiation source and kV detection panel attached to the linac gantry perpendicular to the collimator CAX. The kV images acquired on Elekta linacs are corrected for sag of the kV panel as the gantry rotates using the flexmap,^18^ which is a correction look‐up table for kV panel position. In this study all kV panel centers used to determine the kV imaging isocenter were corrected for the panel sag using the flexmap values included in the DICOM metadata for each kV image. The MV EPID imaging system does not have a correction look‐up table for MV panel position and MV panel center was used as the MV image center without any corrections.

The assessment of isocenters positions alignment was complemented with the corresponding isocenters sizes. The isocenter size in this study was defined as the radius of the sphere comprising all beam CAX for a given energy, or image centers for a given imaging modality.

The comprehensive assessment was performed for all beam energies (6MV, 15MV, 6MV FFF, and 10MV FFF) and imaging modalities (MV and kV).

## 3 RESULTS

An example of the EPID image acquired and processed by the IHD software with the FSO and the BB phantoms is shown in Figs. 2(a) and 2(b) with detected edges of the radiation field, four peripheral BBs, and one central BB.

### 3.1 Localizing collimator CAX at the EPID level

**Fig. 4:**
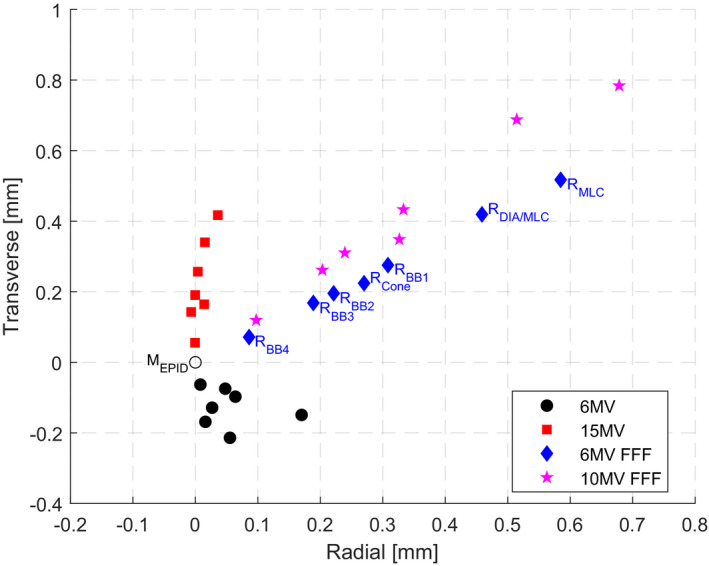
Results for four different energies (6MV, 15MV, 6MV FFF, and 10MV FFF) and five different CAX‐defining systems using BLDs and BBs (the MLC only, the Diaphragm and the MLC, the FSO phantom, the Validation phantom and the Aktina cone) to observe positional relation of the radiation beam CAX (R) and the mechanical collimator CAX (M_EPID_). The radiation beam CAX (R) for different BLDs and BBs are marked only for one 6MV FFF energy for clarity.

**Fig. 5:**
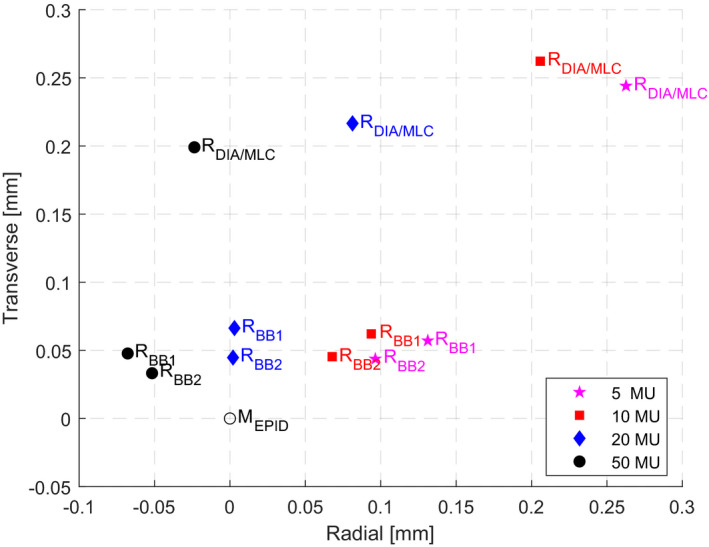
Results of the MU settings (5, 10, 20, and 50) dependency of the radiation beam CAX collimated by the Diaphragms and MLC (R_DIA/MLC_) as well as defined by the BB_1_ and BB_2_ sets (R_BB1_ and R_BB2_, respectively) in relation to the mechanical collimator CAX (M_EPID_) position using the FSO sequence with the 6MV beam and the FSO phantom at gantry 0.

Figure 4 shows convergence of beam CAX, collimated by different BLDs and defined by different BBs, to the collimator CAX for all beam energies. It was observed, as expected, that the closer the position of the BLD or the BB to the linac isocenter, the smaller the BO (due to the non‐zero FSO) measured by the EPID for a given BLD or BB and energy [see the schematic geometry in Fig. 3(b)]. Figure 5 shows convergence of beam CAX, collimated by the diaphragms and the MLC and defined by the FSO phantom (BB_1_ and BB_2_), to the collimator CAX for different MU settings (5, 10, 20, and 50).

Table 1 shows measured FSOs using the FSO and Validation phantoms. It was noted that the FSO for the 6MV beam is slightly different when determined with the FSO phantom (BB_1_ and BB_2_) and the Validation phantom (BB_3_ and BB_4_). It is likely that using the other high energies in between the 6MV exposures affected the FSO of the 6MV beam.

**Table 1:** Results of the focal spot offset (FSO) determined for varying energies (6MV, 15MV, 6MV FFF, 10MV FFF) based on Eq. 2 and using two phantoms (the FSO phantom, the Validation phantom) at gantry angle 0°.

FSO	6MV (mm)	15MV (mm)	6MV FFF (mm)	10MV FFF (mm)
FSO phantom				
Radial	0.07	−0.20	−0.25	−0.41
Transverse	−0.05	−0.01	−0.27	−0.29
Total	0.09	0.20	0.36	0.50
Validation phantom				
Radial	0.15	−0.21	−0.22	−0.35
Transverse	−0.05	0.01	−0.23	−0.22
Total	0.16	0.21	0.32	0.42

Localization of the collimator CAX test showed high accuracy of the mechanical collimator CAX determination (M^E^_EPID_) compared to the radiation beam CAX (R^E^_DIA/MLC_) with the uncertainty measured by varying energy to be in total 0.019 mm and 0.422 mm respectively, and by varying MU (M^MU^_EPID_) and (R^MU^_DIA/MLC_) to be in total 0.003 mm and 0.131 mm respectively (see Table 2).

**Table 2:** Localization uncertainties of the mechanical collimator CAX (M^E^_EPID_), the reference collimator CAX (M^E^_REF_) and the radiation beam CAX (R^E^_DIA/MLC_) for varying energies (6MV, 15MV, 6MV FFF, 10MV FFF) and localization uncertainties of the mechanical collimator CAX (M^MU^_EPID_) and the radiation beam CAX (R^MU^_DIA/MLC_) for varying MUs (5, 10, 20, 50).

Uncertainty (1SD)	M^E^_EPID_ (mm)	M^E^_REF_ (mm)	R^E^_DIA/MLC_ (mm)	M^MU^_EPID_ (mm)	R^MU^_DIA/MLC_ (mm)
Radial	0.08	0.009	0.237	0.002	0.128
Transverse	0.017	0.014	0.349	0.002	0.028
Total	0.019	0.017	0.422	0.003	0.131

### 3.2 Localizing collimator CAX for the BB phantom

**Fig. 6:**
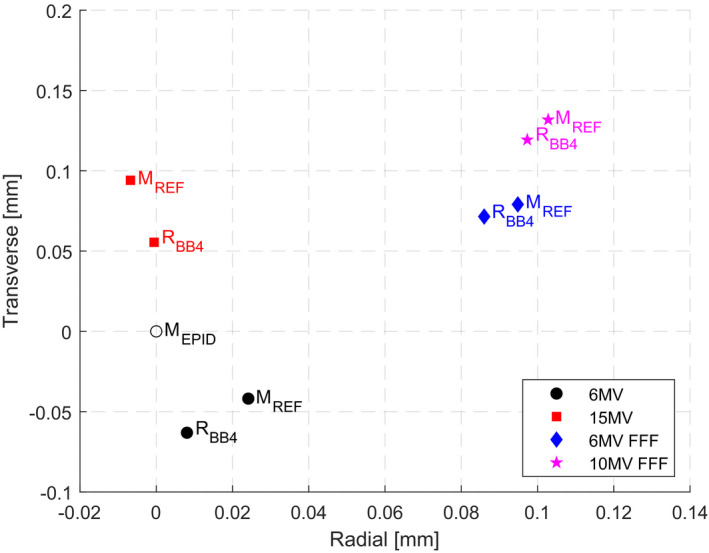
Results of the determination method of the reference mechanical collimator CAX (M_REF_) position against the radiation CAX position defined by the ball‐bearing set BB_4_ (R_BB4_) placed at the linac isocenter using various energies. The mechanical collimator CAX (M_EPID_) measured at the EPID level is also marked in the graph to show its relative position.

Validation of the reference collimator CAX (M^E^_REF_) determination method against the radiation beam CAX location defined by the BB_4_ ball‐bearing set (R^E^_BB4_) placed at the linac isocenter shows negligible variations between energies, on average 0.017 mm in total (see Fig. 6), almost the same determination uncertainty as the collimator CAX (M^E^_EPID_) (see Table 2).

### 3.3 Methods of assessment of linac mechanical, radiation, and imaging isocenters

#### 3.3.1 Coincidence assessment of mechanical and radiation isocenters

An example of coincidence assessment is presented in Table 3 for the 6MV beam in terms of the measured FSO and BO at five gantry angles in radial and transverse directions. Mean and standard deviation of the FSO and the BO should be minimized to achieve congruence of mechanical and radiation isocenters.

**Table 3:** An example of the focal spot offset (FSO) and the beam offset (BO) for the 6MV beam in radial and transverse directions at five gantry angles.

6MV	−180° (mm)	−90° (mm)	0° (mm)	90° (mm)	+180° (mm)	MEAN ± 1SD (mm)
Radial FSO	0.26	0.21	0.07	0.18	0.29	0.20 ± 0.09
Transverse FSO	0.31	0.12	−0.03	0.17	0.27	0.17 ± 0.13
Radial BO	0.56	0.50	0.19	0.34	0.62	0.44 ± 0.18
Transverse BO	0.33	−0.03	0.27	0.03	0.27	0.18 ± 0.16

#### 3.3.2 Stereotactic beam isocentricity assessment

**Fig. 7:**
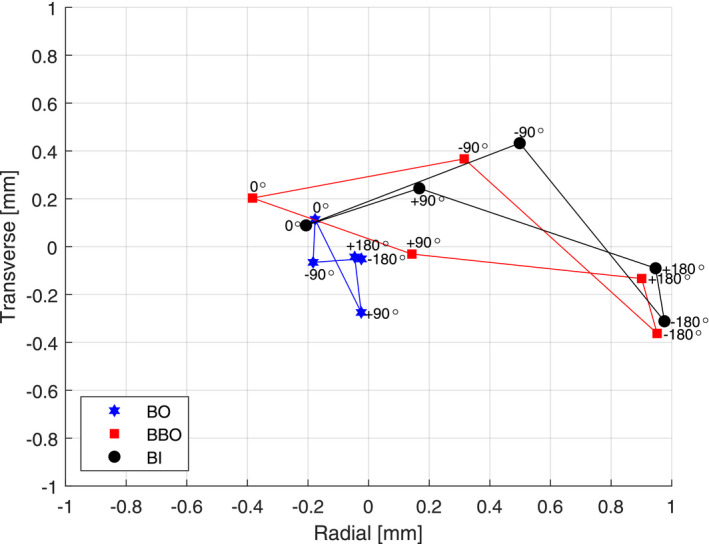
An example of the stereotactic beam isocentricity assessment of the 6MV FFF beam illustrating the beam offset (BO) collimated by the Cone, the BB phantom position deviations (offsets) from the collimator CAX (BBO) and beam isocentricity (BI).

An example of the stereotactic beam isocentricity assessment of the 6MV FFF beam is presented in Fig. 7 and Table 4 in terms of mean values of the beam offset (BO) collimated by the Cone, the BB phantom position deviations (offsets) from the collimator CAX (BBO) and beam isocentricity (BI) in both radial and transverse directions. The beam isocentricity is determined and assessed traditionally as the beam deviations from the BB phantom from the WL test. However, if the test results (BI) are out of tolerance, it is not possible to differentiate whether the root cause of it is the BO, the BBO, or both.

**Table 4:** An example of the stereotactic beam isocentricity assessment for the 6MV FFF beam; mean and one standard deviation of the beam offset (BO) collimated by the Aktina Cone, the ball‐bearing offset (BBO) and the beam CAX deviations from the BB phantom (beam isocentricity ‐ BI) in radial and transverse directions averaged over all gantry and collimator angles.

6MV FFF	BO (mm)	BBO (mm)	BI (mm)
Radial (Mean ± 1SD)	−0.09 ± 0.08	0.39 ± 0.56	0.48 ± 0.50
Transverse (Mean ± 1SD)	−0.06 ± 0.14	0.01 ± 0.29	0.07 ± 0.30

For this particular example, Fig. 7 and Table 4, the measured BBO has a larger uncertainty compared to the BO, indicating larger mechanical collimator CAX deviations from the linac isocenter comparing to radiation beam CAX deviations from the collimator CAX, especially in the radial (longitudinal) direction.

#### 3.3.3 Comprehensive assessment of linac mechanical, radiation, and imaging isocenters

**Fig. 8:**
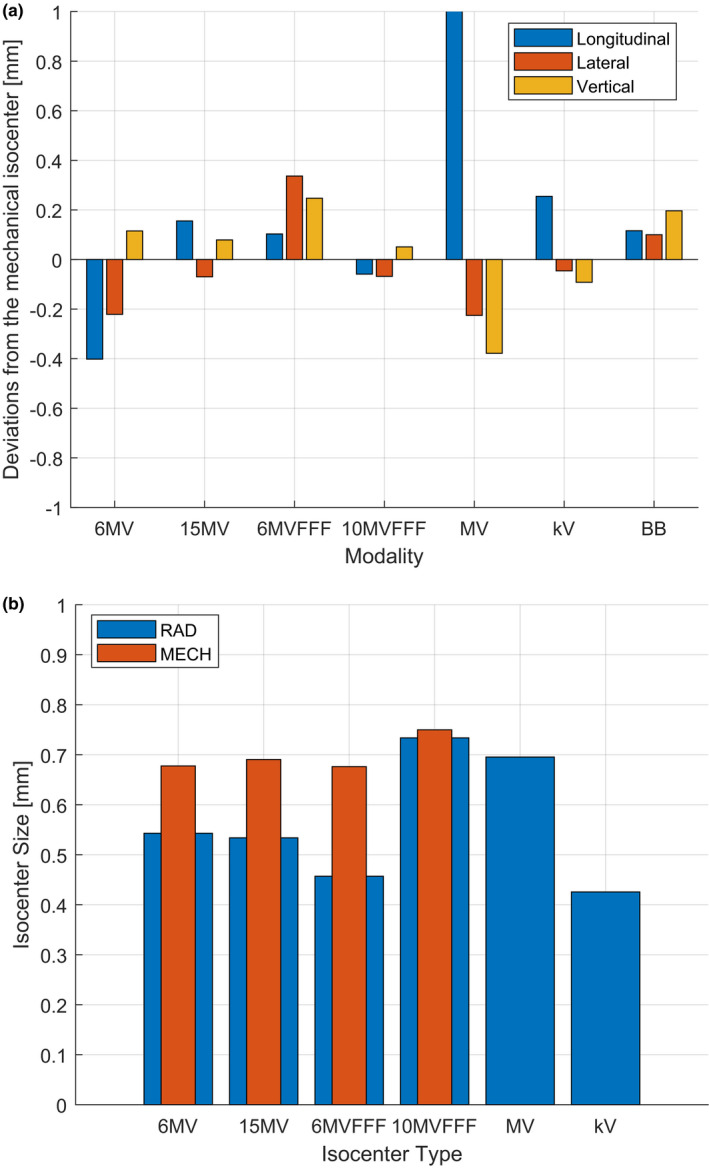
An example of the comprehensive assessment of (a) positions of mechanical, radiation, and imaging isocenters and the BB phantom as well as (b) isocenters sizes.

An example of the comprehensive assessment is presented in Figs. 8(a) and 8(b) for all beam energies (6MV, 15MV, 6MV FFF, and 10MV FFF) and imaging modalities (MV and kV) as well as the BB phantom (BB).

Presenting the results for all isocenters positions or sizes on one graph simplifies the overall assessments of the linac geometrical treatment accuracy and helps in identifying any changes over time thanks to using a stable reference mechanical isocenter position.

All deviations are within the TG142^1^ recommended tolerance of 1 mm for SRS/SBRT techniques, except for the MV EPID panel’s longitudinal position, which indicates that the panel position needs to be recalibrated [see Fig. 8(a)].

The position deviation of the BB phantom from the mechanical isocenter shows the accuracy (of the day) of the image guidance system to align the target with the treatment isocenter, referred to commonly as the IGRT end‐to‐end test.

The uncertainty of the mechanical isocenter position determination (0.05 mm) with varying energies is observed to be significantly smaller than that for the radiation isocenter (0.36 mm).

It is also interesting to see the differences in mechanical and radiation isocenter sizes [see Fig. 8(b) and Table 5]. Radiation isocenter size is dependent on the transverse beam steering and varies from below 0.5 mm for 6MV beam to over 0.7 mm for 10MV FFF beam (1SD = 0.12 mm), whereas mechanical isocenter size is relatively constant, as expected, at 0.7 mm (1SD = 0.04 mm).

**Table 5:** Mean and one standard deviation of the size of mechanical and radiation (collimated by the Aktina Cone and by the diaphragms and the MLC) isocenters for four energies (6MV, 15MV, 6MV FFF, 10MV FFF).

	Mechanical (mm)	Radiation (Aktina cone) (mm)	Radiation (DIA/MLC) (mm)
Isocenter size (Mean ± 1SD)	0.70 ± 0.04	0.64 ± 0.08	0.57 ± 0.12

#### 3.3.4 Isocenters assessment uncertainty analysis

The accuracy of the isocenters assessment methods depends on the accuracy of the EPID measurements and software calculation. The software calculation relies on the correct representation of the experimental model i.e., the geometrical distances of the EPID, linac isocenter, the ball‐bearing sets BB_1_ and BB_2_ from the radiation source (d_EPID_, d_ISO_, d_BB1_, d_BB2_).

Distances of the ball‐bearing sets BB_1_ and BB_2_ from the radiation source (d_BB1_, d_BB2_) are fixed but might be difficult to measure precisely. Therefore, those distances were adjusted in the software model, so the uncertainty of mechanical isocenter position and size determined using four beam energies (i.e., four FSOs) were minimal. The reason for this is that the uncertainty of the mechanical isocenter position and size should not depend on the FSO (i.e., energy). The residual uncertainties related to applied correction factors for d_BB1_ and d_BB2_ are due to the limiting precision of optimizing those distances and are estimated to be ±0.1 mm.

During gantry rotation the distances of the treatment isocenter (d_ISO_) and the EPID (d_EPID_) to the radiation source change, which need to be accounted for by the software. The linac isocenter distance (d_ISO_) average value was not measured and is assumed to be the nominal 100 cm and changes −1.5 mm for gantry angle 0°, +1.5 mm for gantry angle ±180° and no changes for gantry angles ±90°. The EPID distance (d_EPID_) is on average 159 cm (measured based on a known object size placed at the isocenter) and changes +2 mm for gantry angle 0°, −2 mm for gantry angle ±180°, and no changes for gantry angles ±90° (this means that the EPID distance to the linac isocenter changes +3.5 mm and −3.5 mm for gantry angles 0° and 180° respectively). The residual uncertainties related to applied correction factors for d_EPID_ and d_ISO_ are due to the precision of measuring those distances and are estimated to be ±0.5 mm.

### 3.4 Estimated time for proposed methods

The machine access time required to perform the simple coincidence test of mechanical and radiation isocenters for one energy from start to finish takes about 20–30 min, whereas tests focused more on stereotactic treatment accuracy and comprehensive linac assessment take about 40–60 min.

## 4 DISCUSSION

Comprehensive assessment combines many individual recommended tests such as coincidence of mechanical and radiation isocenters, coincidence of treatment and imaging isocenters, isocenters sizes as well as the end‐to‐end IGRT QA test.

The proposed methods are relatively fast and efficient. The basic coincidence assessment procedure consisting of attaching the FSO phantom and delivering the FSO field sequence with one energy, takes about 20–30 min. Determination of the radiation isocenter characteristics is performed using radiation fields defined by MLC and diaphragms and at the same time the mechanical isocenter characteristics can be determined using the FSO phantom. The efficiency comes from the fact that the congruence assessment between those two isocenters is done in a single process automated by the linac hardware and the IHD software.

It may be noted that, of course, the couch positioning, axes, limitations, and deviations are also an integral part of the overall geometric uncertainties of the system for the practical applications considered, e.g., stereotactic treatments. However, these can be assessed separately and are not evaluated here, since we are primarily concerned with the geometric behavior of the linac itself.

In the presented comprehensive assessment example, the uncertainty of determining the mechanical isocenter position and size are 0.05 mm and 0.04 mm, respectively, and the uncertainty of determining the radiation isocenter position and size are 0.36 mm and 0.12 mm respectively. This demonstrates that the applied methodology of localizing the mechanical isocenter is effective and precise compared to the normally used methodology for the radiation isocenter. This is because the localization of the radiation isocenter position (as opposed to the mechanical) depends on the energy, collimation, and MU i.e., is sensitive to adjustments of beam characteristics such as mean photon energy, FSO, start‐up beam stability.

It is recommended that the mechanical isocenter position should be used as the reference linac treatment isocenter. This approach is in line with the recommendation by ‘AAPM Medical Physics Practice Guideline 8.a.: Linear accelerator performance tests’^19^ in which the appropriate method should be established during the linac acceptance/commissioning to define the (linac) isocenter position in an appropriate reference frame that assures required accuracy at the radiotherapy clinic.

The linac isocenter size is a very important linac specification parameter that is used for acceptance testing, especially if the linac is planned to be used for SRS/SBRT treatments. The mechanical isocenter size shows minimal variation with the energy and no variation with the BLD (see Table 5) and is therefore recommended to be used for linac acceptance testing, with other isocenters referenced to it.

Localizing mechanical isocenter position with the use of the BB phantom helps in setting‐up or independently correcting linac radiation and imaging isocenters. Namely, the BB phantom should be positioned at the mechanical isocenter. All radiation isocenter positions can then be adjusted to be aligned with the mechanical isocenter by appropriate beam steering in the radial direction as well as radiation isocenters sizes can be minimized by appropriate beam steering in the transverse direction.

The image guidance system can also be calibrated (or referenced) at the same time using the BB phantom. That methodology would assure congruence for all linac isocenters i.e. mechanical, radiation, and imaging. Moreover, if any of the linac isocenters drifted it can be adjusted accordingly without the need to affect, adjust, or calibrate other isocenters.

The Elekta proprietary procedure for localizing the radiation isocenter, as part of the kilovoltage (kV) imager flexmap calibration process,^18^ uses a predefined field sequence called ‘kV FlexMap Cal. MLC160’. This sequence differs from the FSO sequence, since it uses four gantry angles (instead of five) and 12.5 MU per field (instead of 50 MU). The Elekta sequence designated for localization and alignment assessment, must therefore be used with caution, since low MU per field and lack of data from the fifth gantry angle might substantially alter the radial beam CAX position for each gantry angle, affecting longitudinal isocenter localization.

In the hypothetical scenario where the FSO is zero and there is no MLC or diaphragm sag at any gantry angle it would result in a zero value BO, eliminating the FSO effect, and guaranteeing alignment of mechanical and radiation isocenters. However, this might not be the optimal linac set‐up since the gantry sag and the collimator tilt causes the mechanical and radiation isocenter sizes to be nonzero. Also, treatment planning systems assume that the radiation isocenter size is zero. Therefore, for linacs designated for SRS/SBRT, it is advantageous to correct the transverse beam steering to minimize the radiation isocenter size at the cost of reintroducing a small FSO effect in the transverse direction. A practical guide for optimizing beam steering for SRS/SBRT linacs is the subject of future study.

## 5 CONCLUSION

A new approach for localizing linac collimator CAX and mechanical isocenter using radiation has been presented. The mechanical isocenter position was proven not to depend on the beam energy, the beam collimating device or the beam MU settings, as expected, and it is therefore recommended to be used as a reference treatment isocenter for adjusting radiation and imaging isocenters positions. Also, the mechanical isocenter size parameter was proven not to depend on the beam settings, as expected, as opposed to the radiation isocenter size, and it is therefore recommended to be the standard specification parameter in the linac customer acceptance procedure. The new methodology of localizing mechanical isocenter is efficient and effective and is presented in this study with three complementary practical applications of position and size assessment procedures of mechanical, radiation, and imaging isocenters combined with the patient positioning system.

## CONFLICT OF INTEREST

The authors declare no conflict of interest.

## Data Availability

The data that support the findings of this study are available from the corresponding author upon reasonable request.

## Note

^1^
Radial (R)= Yg, Transverse (T) = Xg (g=gantry coordinate system) Lateral = Xf, Longitudinal = Yf, Vertical = Zf (f=fixed coordinate system), compliant with IEC 1217
